# Real-world comprehensive genomic and immune profiling reveals distinct age- and sex-based genomic and immune landscapes in tumors of patients with non-small cell lung cancer

**DOI:** 10.3389/fimmu.2024.1413956

**Published:** 2024-06-21

**Authors:** Zachary D. Wallen, Heidi Ko, Mary K. Nesline, Stephanie B. Hastings, Kyle C. Strickland, Rebecca A. Previs, Shengle Zhang, Sarabjot Pabla, Jeffrey Conroy, Jennifer B. Jackson, Kamal S. Saini, Taylor J. Jensen, Marcia Eisenberg, Brian Caveney, Pratheesh Sathyan, Eric A. Severson, Shakti H. Ramkissoon

**Affiliations:** ^1^ Labcorp Oncology, Medical Oncology, Durham, NC, United States; ^2^ Duke University Medical Center, Duke Cancer Institute, Department of Pathology, Durham, NC, United States; ^3^ Duke University Medical Center, Duke Cancer Institute, Department of Obstetrics and Gynecology, Durham, NC, United States; ^4^ Fortrea Inc, Medical Oncology, Durham, NC, United States; ^5^ Labcorp, Early Development Laboratories, Burlington, NC, United States; ^6^ Illumina Inc, Medical Oncology, San Diego, CA, United States; ^7^ Wake Forest Comprehensive Cancer Center, Wake Forest School of Medicine, Department of Pathology, Winston-Salem, NC, United States

**Keywords:** cancer, NSCLC, non-small cell lung cancer, tumor microenvironment, inflammation, gene expression, genomics, immune profiling

## Abstract

**Introduction:**

Younger patients with non-small cell lung cancer (NSCLC) (<50 years) represent a significant patient population with distinct clinicopathological features and enriched targetable genomic alterations compared to older patients. However, previous studies of younger NSCLC suffer from inconsistent findings, few studies have incorporated sex into their analyses, and studies targeting age-related differences in the tumor immune microenvironment are lacking.

**Methods:**

We performed a retrospective analysis of 8,230 patients with NSCLC, comparing genomic alterations and immunogenic markers of younger and older patients while also considering differences between male and female patients. We defined older patients as those ≥65 years and used a 5-year sliding threshold from <45 to <65 years to define various groups of younger patients. Additionally, in an independent cohort of patients with NSCLC, we use our observations to inform testing of the combinatorial effect of age and sex on survival of patients given immunotherapy with or without chemotherapy.

**Results:**

We observed distinct genomic and immune microenvironment profiles for tumors of younger patients compared to tumors of older patients. Younger patient tumors were enriched in clinically relevant genomic alterations and had gene expression patterns indicative of reduced immune system activation, which was most evident when analyzing male patients. Further, we found younger male patients treated with immunotherapy alone had significantly worse survival compared to male patients ≥65 years, while the addition of chemotherapy reduced this disparity. Contrarily, we found younger female patients had significantly better survival compared to female patients ≥65 years when treated with immunotherapy plus chemotherapy, while treatment with immunotherapy alone resulted in similar outcomes.

**Discussion:**

These results show the value of comprehensive genomic and immune profiling (CGIP) for informing clinical treatment of younger patients with NSCLC and provides support for broader coverage of CGIP for younger patients with advanced NSCLC.

## 1 Introduction

Lung cancer is the leading cause of cancer-related mortality worldwide, accounting for 1.8 million deaths globally in 2020. More than 238,000 new cases of lung cancer were estimated for 2023 in the United States (US), and 85% of cases will be diagnosed as non-small cell lung cancer (NSCLC) (1). The median age for diagnosis of NSCLC is 70 years, however, approximately 5% are detected in younger patients, between the ages of 40 to 50 years (2, 3). Younger patients with NSCLC represent a clinically significant patient population with distinct clinicopathological features. Further, younger patients have higher prevalence of adenocarcinoma histology, are more likely to be females and never-smokers and are more likely to present with advanced disease at the time of diagnosis compared to older patients (4–8). At the genomic-level, younger patient tumors have been shown to be enriched for clinically actionable genomic alterations including certain driver-mutations in *EGFR*, *ALK*, *ERBB2*, and *ROS1* and deficient in *MET* exon 14 skipping mutations compared to older patient tumors (3, 6, 7, 9–13). Younger patients have tumors with lower tumor mutational burden (TMB) suggesting they may have reduced responses to immunotherapy compared to older patients (7, 14). Younger patient tumors have also been shown to have differentially expressed immune-related genes compared to older patient tumors (14), although more needs to be done to assess the importance of this finding in NSCLC. It remains unclear whether younger patients have better, or worse prognosis or outcomes compared to older patients as previous studies have shown conflicting results (8, 10, 11, 15–21). Response to immunotherapy may vary by age in NSCLC, but inter-study heterogeneity make it unclear whether age independently impacts outcomes (22–26).

Although much has been described about the landscape of clinicopathological and genomic features in younger patients with NSCLC, this population of patients remains understudied (3). Previously reported studies of clinicopathological features, genomic alterations, and/or treatment outcomes in younger NSCLC have also shown inconsistent results. This may be due to differences in the underlying study population (i.e., differences in sex, age, race/ethnicity), treatments provided, or methodological strategies such as the age threshold at which a study defines younger and older patients. Additionally, few studies have incorporated sex into their analyses via covariate adjustment or stratification (6, 8, 10, 11, 15–21); an important consideration as sex differences have been observed for frequencies of certain genomic alterations (e.g., *RBM10*, *STK11*), the tumor immune microenvironment (e.g., inflammation, T-cell infiltration, immune checkpoint molecules), and response to immunotherapy (27). Studies targeting age-related differences in the tumor immune microenvironment are also lacking, which could provide insight into responses, or lack thereof, to immunotherapy in younger patients. Finally, few studies to date have investigated differences in genomic alteration prevalence, tumor immune microenvironment, and responses to immunotherapy congruently within the same study. This could help create a more complete view of the genomic and immune landscape in younger patient tumors and help determine how this may influence response to immunotherapy.

To further elucidate differences between younger and older patients with NSCLC in a real-world clinical setting, we comprehensively characterized both the landscape of genomic alterations and the tumor immune microenvironment in patient tumors tested via comprehensive genomic and immune profiling (CGIP) during standard care, accounting for differences between male and female patients. We defined older and younger patient groups based on the qualification age for the US-based Medicare insurance program (≥65 years). This threshold was selected because it has real-world consequences for patients with NSCLC. Younger, pre-Medicare aged patients typically have less coverage for CGIP compared to Medicare-insured patients (28), even though they may have higher success of being matched to a targeted therapy through CGIP. We further divided younger patients into sub-groups using a 5-year sliding threshold from <45 to <65 years to assess varying levels of younger patients and increase the comprehensiveness of our analysis. Additionally, using an independent cohort of patients with immunotherapy outcomes data, we tested the combinatorial effect of age and sex on overall and progression-free survival of patients with NSCLC given immunotherapy or combination chemoimmunotherapy to assess the clinical value of CGIP for informing clinical treatment of younger patients.

## 2 Methods

### 2.1 Patient cohorts

Approval for this study was obtained from the Western Institutional Review Board Copernicus Group (WCG protocol # 1340120) including waiver of informed consent.

We retrospectively analyzed real-world CGIP testing data from NSCLC FFPE tissue specimens that were submitted for CGIP testing by a reference laboratory (OmniSeq/Labcorp, Buffalo, NY, USA) as part of standard of care from 2021 to 2024. Any cases that were ultra-hypermutated (TMB >200 mutations/Mb) and did not have available age data were excluded from study. The total number of cases included in study was 8,230 cases (4,938 adenocarcinoma, 1,843 squamous cell carcinoma, 64 large cell neuroendocrine and 1,385 other or not otherwise specified NSCLC; **Table 1**) and each case represented a single, distinct data point. Patients were labeled as younger, pre-Medicare aged (<65 years, N=2,111, mean age= 58 ± 6.2) or older, Medicare age-qualified (≥65 years, N=6,119, mean age=75 ± 6.5) patients (**Table 1**). A 5-year sliding threshold from <45 to <65 years was used to define various age groups within the younger, pre-Medicare patient group for statistical analyses (**Table 1**).

**Table 1:** Patient demographics and clinical characteristics of the discovery cohort.

Characteristic	All patients		Age ≥65		Age <65		Age <60		Age <55		Age <50		Age <45		*P* for trend
	N	Summary stats	N	Summary stats	N	Summary stats	N	Summary stats	N	Summary stats	N	Summary stats	N	Summary stats	
**Total number of patients**	8230	–	6119	–	2111	–	989	–	434	–	189	–	100	–	–
**Sex (N, %)**	8226		6117		2109		989		434		189		100		
Female		4131 (50.2%)		3036 (49.6%)		1095 (51.9%)		534 (54%)		228 (52.5%)		95 (50.3%)		48 (48%)	
Male		4095 (49.8%)		3081 (50.4%)		1014 (48.1%)		455 (46%)		206 (47.5%)		94 (49.7%)		52 (52%)	0.07
**Age (Mean ± SD)**	8230	70.5 ± 9.8	6119	74.8 ± 6.5	2111	58 ± 6.2	989	53.3 ± 6.1	434	48.1 ± 6	189	42.9 ± 5.6	100	38.9 ± 4.9	–
**Histology group (N, %)**	8230		6119		2111		989		434		189		100		
Adenocarcinoma		4938 (60%)		3634 (59.4%)		1304 (61.8%)		617 (62.4%)		284 (65.4%)		133 (70.4%)		69 (69%)	0.01
Squamous cell carcinoma		1843 (22.4%)		1464 (23.9%)		379 (18%)		149 (15.1%)		47 (10.8%)		14 (7.4%)		7 (7%)	4E-13
Large cell neuroendocrine		64 (0.8%)		37 (0.6%)		27 (1.3%)		7 (0.7%)		2 (0.5%)		1 (0.5%)		1 (1%)	0.41
Other NSCLC		1385 (16.8%)		984 (16.1%)		401 (19%)		216 (21.8%)		101 (23.3%)		41 (21.7%)		23 (23%)	9E-5
**Tissue specimen site (N, %)**	8230		6119		2111		989		434		189		100		
Primary		5627 (68.4%)		4311 (70.5%)		1316 (62.3%)		594 (60.1%)		243 (56%)		103 (54.5%)		54 (54%)	9E-11
Advanced		1627 (19.8%)		1147 (18.7%)		480 (22.7%)		241 (24.4%)		111 (25.6%)		49 (25.9%)		29 (29%)	1E-4
Metastatic		976 (11.9%)		661 (10.8%)		315 (14.9%)		154 (15.6%)		80 (18.4%)		37 (19.6%)		17 (17%)	8E-6
**Unknown clinical stage (N, %)**		5331 (64.8%)		3969 (64.9%)		1362 (64.5%)		630 (63.7%)		277 (63.8%)		119 (63%)		60 (60%)	–
**Known clinical stage (N, %)**		2899 (35.2%)		2150 (35.1%)		749 (35.5%)		359 (36.3%)		157 (36.2%)		70 (37%)		40 (40%)	–
**Known clinical stage (N, %)**	2899		2150		749		359		157		70		40		
Stage I		313 (10.8%)		250 (11.6%)		63 (8.4%)		25 (7%)		7 (4.5%)		2 (2.9%)		0 (0%)	7E-4
Stage II		179 (6.2%)		135 (6.3%)		44 (5.9%)		15 (4.2%)		2 (1.3%)		0 (0%)		0 (0%)	0.02
Stage III		481 (16.6%)		359 (16.7%)		122 (16.3%)		60 (16.7%)		23 (14.6%)		13 (18.6%)		9 (22.5%)	0.96
Stage IV		1926 (66.4%)		1406 (65.4%)		520 (69.4%)		259 (72.1%)		125 (79.6%)		55 (78.6%)		31 (77.5%)	1E-3
**Number of detected alterations (Mean ± SD)**	6044	4 ± 2.4	4484	4 ± 2.4	1560	4 ± 2.4	726	3.9 ± 2.4	327	3.7 ± 2.3	144	3.5 ± 2.2	76	3.4 ± 2.2	0.01
**Genomic alterations with known or potential clinical significance (N, %)**	6044		4484		1560		726		327		144		76		
Guideline-indicated		2847 (47.1%)		2106 (47%)		741 (47.5%)		359 (49.4%)		177 (54.1%)		89 (61.8%)		46 (60.5%)	0.37
Clinical trial or therapy in other tumor type		5241 (86.7%)		3884 (86.6%)		1357 (87%)		629 (86.6%)		277 (84.7%)		126 (87.5%)		64 (84.2%)	0.35
Neither above, but known pathogenic		728 (12%)		544 (12.1%)		184 (11.8%)		90 (12.4%)		45 (13.8%)		15 (10.4%)		11 (14.5%)	0.3

Full results including testing results for each individual age group vs ≥65 can be found in **Supplementary Table 1**. Tests for “Number of detected alterations” and “Genomic alterations with known or potential clinical significance” included only patient tumor data that passed sequencing for single nucleotide variants (N=7,179), copy number variants (N=7,291), and fusion/exon skipping variants (N=6,304), and results have been adjusted for tissue specimen site and histology, which showed significant differences between age groups and were used as covariates for downstream analyses. SD, standard deviation of the mean; P for trend, P-value from standard or penalized likelihood ratio test used to test for association of decreasing age with the variable of interest (see Methods). Note, when testing for trends between decreasing age and variables of interest, each patient makes up a distinct data point in the analysis as age is being treated as a quantitative variable.

An independent, real-world retrospective cohort of 250 NSCLC patients treated with pembrolizumab immunotherapy (N=102) or pembrolizumab immunotherapy with chemotherapy (N=148) that had CGIP testing completed by a reference laboratory (OmniSeq/Labcorp, Buffalo, NY, USA) during standard clinical care from 2017 to 2021 was used to test the association of age with overall survival (OS) and progression-free survival (PFS) of patients. Like the main discovery cohort, most patient tumors had non-squamous histology (N=213, 85%) and relatively equal distribution of males and females (46% and 54%, respectively). Patients were labeled as younger, pre-Medicare aged (<65 years, N=90, mean age=58 ± 5.3) or older, Medicare age-qualified (≥65 years, N=160, mean age=73 ± 6.2), however, the younger, pre-Medicare age group was not further split into smaller sub-groups due to sample size.

### 2.2 Comprehensive genomic and immune profiling

DNA and RNA were co-extracted from FFPE tissue specimens and submitted for library preparation and DNA and RNA sequencing using OmniSeq^®^ INSIGHT (OmniSeq/Labcorp, Buffalo, NY, USA) (29). OmniSeq^®^ INSIGHT is a next generation sequencing-based, laboratory-developed test for the detection of genomic variants, signatures, and immune gene expression in FFPE tumor tissue, performed in a laboratory accredited by the College of American Pathologists (CAP) and certified by the Clinical Laboratory Improvement Amendments (CLIA). DNA sequencing with the hybrid-capture-based TruSight^®^ Oncology 500 assay (Illumina, San Diego, CA, USA) is used to detect small variants in the full exonic coding region of 523 genes (single and multi-nucleotide substitutions, insertions, and deletions), copy number alterations in 59 genes (gains and losses), microsatellite instability and TMB genomic signatures. RNA sequencing with hybrid-capture is used to detect fusions and splice variants in 55 genes. Gene expression of 397 immune-related genes is interrogated via an amplicon-based targeted RNA sequencing assay (Oncomine™ Immune Response Research Assay, ThermoFisher, Waltham, MA, USA). Of these genes, expression of 64 currently have clinical evidence supporting their potential as immunotherapy targets and have been previously validated for monitoring immune activity in solid tumors (30, 31). Of note, while the same assay was used to profile immune gene expression of patient tumors in the independent cohort with clinical outcomes, an earlier, smaller assay (OmniSeq^®^ Comprehensive, 144 gene panel) was used to detect genomic alterations.

### 2.3 Marker clinical significance

The criteria used to classify the clinical significance of reported genomic variants for the OmniSeq^®^ INSIGHT CGIP assay relative to the tested tumor type is reported in accordance with AMP/CAP/ASCO guidelines for the interpretation and reporting of sequence variants in cancer (32). While this guidance was developed specifically for genomic variants, OmniSeq^®^ INSIGHT extends interpretation and application of this classification to all reported markers including immune gene expression markers for response to immunotherapy. Tier I genomic alterations or immunotherapy markers have strong clinical significance with FDA-approved or professional guideline-indicated therapies for the tested tumor type. Tier II genomic alterations or immunotherapy markers have potential clinical significance including FDA-approved therapies for other tumor types, clinical trial inclusion criteria for the tested tumor type, other evidence for plausible therapeutic significance in the tested tumor type or are known pathogenic variants. Only detected pathogenic genomic alterations that were classified as clinically significant or potentially clinically significant were included in this study.

### 2.4 Gene expression bioinformatic processing

Absolute read counts for each gene transcript were generated using the Ion Torrent Suite Software plugin immuneResponseRNA (ThermoFisher, Waltham, MA), then background read counts from a sequenced no template control sample were subtracted to produce background subtracted read counts (BSRC). Per sample normalized reads per million (nRPM) values were then calculated as previously described to make RNA-seq measurements across runs and samples comparable (30). Briefly, sample BSRCs are normalized by obtaining sample-to-control ratios of 10 housekeeping gene BSRCs compared against a pre-constructed housekeeping gene RPM profile from an external, validated control sample, and the median ratio is used as a normalization ratio for the sample. Then, for each transcript, sample BSRCs are divided by the normalization ratio to obtain nRPM values. From the nRPM values, normalized gene expression ranks are calculated as a percentile rank from 1 to 100 by comparing nRPM values to those of a pan-cancer reference population derived from 735 unique tumors. Tumors can then be classified as “high expressors” of a particular gene if a gene’s normalized gene expression rank reaches 75 or above. Three immune gene expression signatures are calculated from the gene expression ranks: tumor immunogenic score (TIGS) (33), cellular proliferation (CP) score (34), and cancer testis antigen burden (CTAB) score (35). The average (for TIGS and CP) or the sum (for CTAB) of gene expression rankings of previously identified signature-specific sets of genes are taken as the score for each signature. These immune gene expression signatures are calculated to capture information about the tumor immune microenvironment including inflammation of the tumor tissue (TIGS), proliferation of tumor and/or immune cells (CP), and presence of cancer testis antigens (CTAB), all of which have previously been shown to help predict outcome for immune checkpoint inhibition therapies in conjunction with traditional immunotherapy biomarkers (33–35).

### 2.5 Immunohistochemical studies

PD-L1 expression on the surface of tumor cells was measured by Dako^®^ PD-L1 IHC 22C3 pharmDx (Agilent, Santa Clara, CA). Expression was scored by a board-certified anatomical pathologist according to published guidelines (36) as tumor proportion score (TPS), which is the percentage of tumor cells with positive linear membranous staining.

### 2.6 Statistical analysis

Statistical analysis and plotting were performed in R v 4.2.2 (https://www.r-project.org/). All plotting was performed using ggplot2 v 3.4.0 (https://ggplot2.tidyverse.org/) and various packages to extend the ggplot2 functionality (ggfortify, ggpubr, ggtext, plotly, GGally, ggrepel). All statistical analyses detailed below used the ≥65 age group as the reference to derive coefficients that described the change observed in younger NSCLC patient tumors as compared to older patient tumors. All reported *P*-values are two-sided.

To test for differences in the frequencies of patient demographic variables (biological sex) and clinical characteristics (clinical stage, histology, tumor specimen site) between younger age groups and the ≥65 age group, Fisher’s exact test was performed via the `fisher.test` function. Additionally, differences in number of genomic alterations detected and the frequency of detected genomic alterations with known or potential clinical significance was tested using linear or Firth’s penalized logistic regression, respectively, via the `lm` function or `logistf` function (from logistf v 1.24.1, https://cran.r-project.org/web/packages/logistf) adjusting for histology (adenocarcinoma, squamous cell carcinoma, large cell neuroendocrine, other, or not otherwise specified NSCLC) and tumor specimen site (primary, advanced, metastatic).

To test for differences in genomic alterations, immunotherapy markers, immunogenic signatures, and immune gene expression between younger age groups and the ≥65 age group, linear or Firth’s penalized logistic regression was performed for quantitative and categorical variables, respectively, adjusting for tumor specimen site and histology. To test for any trends in results, two linear or Firth’s logistic regression models were tested: (1) testing association of age (treated as a quantitative variable with ages ≥65 collapsed to 65) with the feature of interest and (2) an age-null model where age was not included, only non-age covariates. Then, the two models were compared for significant differences in fits by performing a standard (for linear regression) or penalized (for Firth’s logistic regression) likelihood ratio test using the `anova` function. Note, when testing for differences between younger age groups and the ≥65 age group, data for a patient may appear in multiple age groups (e.g., a 50-year-old patient will be present in the <65, <60, and <55 age groups, but not the <50 and <45 age groups). However, when testing trends between decreasing age and variables of interest, each patient makes up a distinct data point in the analysis as age is being treated as a quantitative variable.

TMB, the immune signature scores CP and CTAB, and nRPM gene expression values were log transformed (after pseudo-count of 1 added) prior to testing due to the data being heavily skewed toward zero. *P*-values from testing genomic alterations and individual immune gene expression were multiple testing corrected using the Benjamini-Hochberg false discovery rate (FDR) method as implemented in the `p.adjust` function. Thresholds for significance were set at <0.05 for uncorrected *P*-values and <0.25 for multiple testing corrected *P*-values. The <0.25 threshold is commonly used threshold for *P*-values that have been multiple testing corrected using the FDR procedure and when testing large numbers of features such as in pathway enrichment (37) and microbiome analysis (38). When testing differences in prevalence of genomic alterations, analyses were performed twice: once aggregating all single nucleotide variants (SNVs), copy number variants (CNVs), and fusions/rearrangements at the gene-level (gene-level genomic alterations), and again, testing individual SNVs, CNVs, and fusions/rearrangements. Gene-level alterations and individual variants detected in at least 5 patient tumors were included in testing.

To test if younger NSCLC patients had significantly better or worse outcomes on pembrolizumab immunotherapy or pembrolizumab immunotherapy with chemotherapy than older NSCLC patients, Cox proportional hazards regression was performed adjusting for histology, and Kaplan-Meier curves generated via the `coxph` and `survfit` functions from survival v 3.4–0 (https://cran.r-project.org/web/packages/survival). Cox regression was performed twice, once testing differences in OS and PFS between patients <65 years and ≥65 years, and again testing for any trends in association of survival with decreasing age by treating age as a quantitative variable with ages ≥65 collapsed to 65. Kaplan-Meier curves were constructed for <65 *vs* ≥65 and plotted using ggplot2. As the cohort with outcomes data was much smaller than that of the main patient cohort, only the largest and most statistically powered age groups (<65 and ≥65) were used for Cox regression and Kaplan-Meier curves. Uncorrected *P*-values <0.05 were considered significant.

Linear, Firth’s and Cox regression analyses were performed for all patients, then for males and females separately. Additionally for immunogenic signatures, immune gene expression, and survival analyses, each analysis was repeated testing differences between males and females overall. To ensure detected associations between age and immunogenic signatures or immune gene expression were not merely due to the presence of underlying *ALK* or *EGFR* genomic alterations, analyses were repeated adding presence or absence of *ALK* or *EGFR* genomic alterations as a covariate to the statistical model.

To determine if differentially expressed gene lists of interest were significantly enriched in particular Gene Ontology (GO) biological processes, the PANTHER Overrepresentation Test (PANTHER v 18.0) (39, 40) was performed against the PANTHER GO-Slim Biological Process database via the web-based GO Enrichment Analysis interface (http://geneontology.org/) using default parameters.

## 3 Results

### 3.1 Patient demographics and clinical characteristics

This study included data on 8,230 patient tumors submitted for CGIP during standard-of-care testing, with 6,119 aged ≥65 years (74%) and 2,111 aged <65 years (26%). Among the latter, there were 989 patients aged <60 years (12%), 434 aged <55 years (5%), 189 aged <50 years (2%), and 100 aged <45 years (1%). The ratio of females to males were approximately 1:1 when looking at all patients regardless of age (50.2% *vs* 49.8%) and did not change significantly with decreasing age (*P* for trend = 0.07). Adenocarcinoma was the predominant histology group in all age groups, and in young patients, adenocarcinoma accounted for upwards of 70% of cases as age decreased (OR = 1.1 to 1.6, *P* for trend = 0.01). Squamous cell carcinoma was detected at a higher frequency in older patients (24% of patients ≥65 years), while making up only 18% of young cases at the highest frequency and significantly decreasing with age down to 7% at the lowest (OR = 0.2 to 0.7, *P* for trend = 4E-13). Contrarily, cases of other or NOS NSCLC was found at higher frequencies as age decreased (OR = 1.2 to 1.6, *P* for trend = 9E-5). Younger patient tumors were more commonly resected from advanced (e.g., lymph nodes) (OR = 1.3 to 1.8, *P* for trend = 1E-4) or metastatic (i.e., distant sites such as brain and liver) (OR = 1.4 to 2, *P* for trend = 8E-6) tissue sites. Younger patient tumors had significantly fewer total pathogenic alterations detected on average compared to older patient tumors (mean=3 ± 2 at lowest *vs* 4 ± 2, *P* for trend = 0.01), yet had similar frequencies of genomic alterations with FDA-approved targeted therapies or clinical trial for NSCLC or an approved therapy in another tumor type (*P* for trend > 0.3). These findings indicated certain patient clinical characteristics (histology and tumor specimen site) varied by age, and therefore, were considered potential confounders and used as covariates for downstream analyses of genomic and immune-related molecular data.

An overview of patient demographics and clinical characteristics are provided in **Table 1**, and odds ratios (OR) and *P*-values for testing each individual age group with the ≥65 age group are presented in **Supplementary Table 1**.

### 3.2 Gene-level genomic alterations and individual variants with differing prevalence between younger and older patient tumors

**Figure 1:**
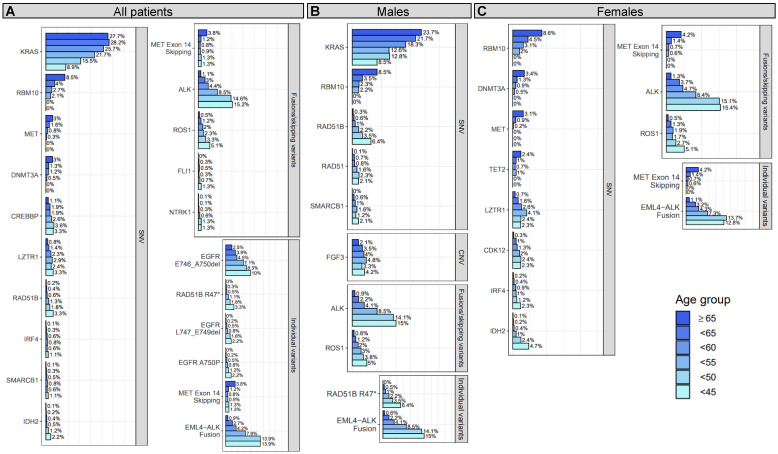
Prevalence of gene-level genomic alterations and individual variants with significant age associations in all, male, and/or female patients with NSCLC. For all patients **(A)**, males **(B)**, and females **(C)**, differences between younger and older patients with NSCLC were assessed for prevalence of genomic alterations at the gene-level and for individual variants. Genomic alterations that showed a significant trend (adjusted *P* for trend <0.25) of decreasing or increasing prevalence with age are shown in the figure. Each bar corresponds to an age group and the length of each bar corresponds to the prevalence (x-axis) of the genomic alteration (y-axis) within that age group. The prevalence is provided at the end of each bar and denotes the percentage of patient tumors a genomic alteration was detected in out of all the patient tumor samples that passed the single nucleotide variant (SNV; N=7,179), copy number variant (CNV; N=7,291), or fusion/skipping variant (N=6,304) component of the genomic profiling assay.

In total, 24 gene-level genomic alterations (defined in Methods) and individual genomic variants were detected as having significantly different prevalence between younger and older patient tumors, the majority of which (75%) had higher prevalence as age decreased (**Figure 1A**; **Table 2**; **Supplementary Tables 2A**, **3A**). More than a third (38%) were genes or specific variants that have known clinical significance in NSCLC, the majority of which (67%), were enriched in younger patient tumors. The remaining alterations included specific variants or genes with variants that have potential clinical significance in NSCLC (known pathogenic, available clinical trial, approved therapy in another tumor type), the majority of which (80%), again, were enriched in younger patient tumors.

**Table 2:** Associations between decreasing age and prevalence of genomic alterations in the discovery cohort.

Alteration	Tier 1 or 2	Enriched age group	Oldest age group first detected for	Patient group detected in	All patients N (%)	Age ≥65 N (%)	Age <65 N (%)	Age <60 N (%)	Age <55 N (%)	Age <50 N (%)	Age <45 N (%)	*P* for trend
Single nucleotide variants												
*KRAS* [G]	1	older	<55	all, male	1998 (27.8%)	1479 (27.7%)	519 (28.2%)	219 (25.7%)	82 (21.7%)	26 (15.5%)	8 (8.9%)	3E-3
*RBM10* [G]	2	older	<65	all, male, female	530 (7.4%)	456 (8.5%)	74 (4%)	23 (2.7%)	8 (2.1%)	0 (0%)	0 (0%)	1E-12
*MET* [G]	1	older	<65	all, female	187 (2.6%)	158 (3%)	29 (1.6%)	7 (0.8%)	1 (0.3%)	0 (0%)	0 (0%)	2E-4
*DNMT3A* [G]	2	older	<65	all, female	184 (2.6%)	160 (3%)	24 (1.3%)	10 (1.2%)	2 (0.5%)	0 (0%)	0 (0%)	3E-3
*CREBBP* [G]	–	younger	<65	all	93 (1.3%)	58 (1.1%)	35 (1.9%)	16 (1.9%)	10 (2.6%)	6 (3.6%)	3 (3.3%)	0.16
*TET2* [G]	–	older	<60	female	73 (1%)	63 (2.4%)	10 (1%)	3 (0.7%)	2 (1%)	0 (0%)	0 (0%)	0.24
*LZTR1* [G]	–	younger	<60	all, female	70 (1%)	44 (0.8%)	26 (1.4%)	20 (2.3%)	11 (2.9%)	4 (2.4%)	3 (3.3%)	0.12
*RAD51B* [G]	2	younger	<55	all, male	19 (0.3%)	12 (0.2%)	7 (0.4%)	5 (0.6%)	5 (1.3%)	3 (1.8%)	3 (3.3%)	0.01
*CDK12* [G]	2	younger	<65	female	17 (0.2%)	7 (0.3%)	10 (1%)	6 (1.3%)	4 (2%)	2 (2.4%)	1 (2.3%)	0.16
*IRF4* [G]	–	younger	none	all, female	14 (0.2%)	8 (0.1%)	6 (0.3%)	5 (0.6%)	3 (0.8%)	1 (0.6%)	1 (1.1%)	0.23
*SMARCB1* [G]	2	younger	<55	all, male	12 (0.2%)	6 (0.1%)	6 (0.3%)	4 (0.5%)	3 (0.8%)	1 (0.6%)	1 (1.1%)	0.13
*IDH2* [G]	2	younger	<50	all, female	9 (0.1%)	6 (0.1%)	3 (0.2%)	3 (0.4%)	2 (0.5%)	2 (1.2%)	2 (2.2%)	0.15
*RAD51* [G]	2	younger	<65	male	8 (0.1%)	2 (0.1%)	6 (0.7%)	3 (0.8%)	3 (1.6%)	2 (2.3%)	1 (2.1%)	0.24
*EGFR* E746_A750del [V]	1	younger	<55	all	207 (2.9%)	136 (2.5%)	71 (3.9%)	38 (4.5%)	27 (7.1%)	14 (8.3%)	9 (10%)	0.05
*RAD51B* R47* [V]	2	younger	<60	all, male	7 (0.1%)	2 (0%)	5 (0.3%)	4 (0.5%)	4 (1.1%)	3 (1.8%)	3 (3.3%)	4E-4
*EGFR* L747 E749del [V]	1	younger	<60	all	5 (0.1%)	1 (0%)	4 (0.2%)	4 (0.5%)	3 (0.8%)	3 (1.8%)	2 (2.2%)	0.01
*EGFR* A750P [V]	2	younger	<60	all	5 (0.1%)	1 (0%)	4 (0.2%)	4 (0.5%)	3 (0.8%)	2 (1.2%)	2 (2.2%)	0.02
Copy number variants												
*FGF3* [G]	2	younger	<65	male	88 (1.2%)	56 (2.1%)	32 (3.5%)	16 (4%)	9 (4.8%)	3 (3.3%)	2 (4.2%)	0.25
Fusions/exon skipping variants												
*MET* Exon 14 Skipping [G/V]	1	older	<65	all, female	196 (3.1%)	176 (3.8%)	20 (1.2%)	6 (0.8%)	3 (0.9%)	2 (1.3%)	1 (1.3%)	3E-5
*ALK* [G]	1	younger	<65	all, male, female	100 (1.6%)	51 (1.1%)	49 (3%)	34 (4.4%)	29 (8.5%)	22 (14.6%)	12 (15.2%)	6E-13
*ROS1* [G]	1	younger	<65	all, male, female	45 (0.7%)	25 (0.5%)	20 (1.2%)	15 (2%)	8 (2.3%)	5 (3.3%)	4 (5.1%)	6E-3
*FLI1* [G]	–	younger	<65	all	5 (0.1%)	0 (0%)	5 (0.3%)	4 (0.5%)	1 (0.3%)	1 (0.7%)	1 (1.3%)	0.15
*NTRK1* [G]	1	younger	<50	all	5 (0.1%)	3 (0.1%)	2 (0.1%)	2 (0.3%)	2 (0.6%)	2 (1.3%)	1 (1.3%)	0.11
*EML4-ALK* Fusion [V]	1	younger	<65	all, male, female	85 (1.3%)	40 (0.9%)	45 (2.7%)	32 (4.2%)	27 (7.9%)	21 (13.9%)	11 (13.9%)	2E-13

Significant associations between decreasing age and prevalence of genomic alterations (P for trend < 0.25) are shown here. Full results, including testing results for each individual age group vs ≥65, can be found in **Supplementary Tables 2**, **3**. Tests for single nucleotide variants, copy number variants, and fusions/exon skipping variants included only patient tumor data that passed sequencing for the corresponding component of the assay (SNV N=7,179, CNV N=7,291, fusion/skipping variant N=6,304). Genomic alterations detected in at least 5 patient tumors were included in the analysis. All results have been adjusted for tumor specimen site and histology. Results shown are from testing all patients unless an association was only detected in males or females, then results are from the corresponding analysis. If “none” is listed for “Oldest age group first detected for”, then association was only detected for P of trend and not any individual age group comparisons. [G] Result from gene-level analysis; [V] Result from analysis of individual variants; [G/V] Same designation and in both gene-level and individual variant analyses (lowest P for trend shown); Tier, denotes if a gene contains Tier 1 and/or 2 alterations or if a particular variant is a Tier 1 and/or 2 alteration as defined by AMP/CAP/ASCO joint guidelines; Tier 1, strong clinical significance in patient’s tumor type (FDA-approved targeted therapy available); Tier 2, potential clinical significance in patient’s tumor type (clinically significant in another tumor type, involved in clinical trial, emerging biomarker, known pathogenic); P for trend, multiple testing corrected P-value from standard or penalized likelihood ratio test used to test for association of decreasing age with the variable of interest (see Methods). Note, when testing for trends between decreasing age and variables of interest, each patient makes up a distinct data point in the analysis as age is being treated as a quantitative variable.

#### 3.2.1 Genomic alterations with established clinical significance in NSCLC

Genomic alterations with established clinical significance were detected as enriched or depleted in younger patient tumors at the gene- and individual variant-level across several genes and two alteration types including SNVs and fusions/exon skipping variants (**Figure 1A**; **Table 2**; **Supplementary Tables 2A**, **3A**). Gene-level genomic alterations with the highest prevalence in younger patients were fusions with *ALK*, which were observed more frequently with decreasing age (1.1% to 15.2%, adjusted *P* for trend = 6E-13). This association was largely driven by the specific *EML4-ALK* fusion, whose frequency increased with decreasing age (0.9% to 13.9%, adjusted *P* for trend = 2E-13). Other gene-level genomic alterations found significantly enriched in younger patient tumors included less prevalent fusions with *ROS1* (5.1% at highest *vs* 0.5% in ≥65 group, adjusted *P* for trend = 6E-3) and *NTRK1* (1.3% at highest *vs* 0.1% in ≥65 group, adjusted *P* for trend = 0.11). Additionally, the prevalence of two specific *EGFR* deletions, E746-A750 (2.5% to 10%, adjusted *P* for trend = 0.05) and L747-E749 (<1% to 2.2%, adjusted *P* for trend = 0.01), showed significant increases with decreasing age. *KRAS* SNVs (most prevalent of which was *KRAS* G12C, 11%) had the highest prevalence in older patient tumors and showed an obvious decrease in prevalence with decreasing age (27.7% to 8.9%, adjusted *P* for trend=3E-3). Prevalence of *MET* exon 14 skipping mutations was 3-fold lower in the <65 age group compared to the ≥65 group (OR = 0.31, adjusted *P* = 1E-6) with only 20 out of 196 (10%) being detected in patients <65 years. Prevalence of *MET* SNVs were even more dramatically reduced in younger patient tumors with a 2-fold lower prevalence in the <65 age group compared to the ≥65 group (OR = 0.52, adjusted *P* = 0.03) and none being detected in patients <50 years.

#### 3.2.2 Genomic alterations with potential clinical significance in NSCLC

Most of the detected genomic alterations with potential clinical significance in NSCLC were found significantly enriched in younger patient tumors including SNVs in *RAD51B, SMARCB1, IDH2, RAD51, CREBBP*, *LZTR1*, *IRF4* and fusions with *FLI1*, while SNVs in *RBM10* and *DNMT3A* were significantly enriched in older patient tumors (**Figure 1A**; **Table 2**; **Supplementary Tables 2A**, **3A**). SNVs in *RAD51B* (3.3% at highest *vs* 0.2% in ≥65 group, adjusted *P* for trend = 0.01), *LZTR1* (3.3% at highest *vs* 0.8% in ≥65 group, adjusted *P* for trend = 0.12), and *CREBBP* (3.3% at highest *vs* 1.1% in ≥65 group, adjusted *P* for trend = 0.16), and the specific *RAD51B* substitution R47* (3.3% at highest *vs <*0.1% in ≥65 group, adjusted *P* for trend = 4E-4) were the most prevalent in younger patient tumors. The specific *EGFR* substitution A750P had the next highest prevalence in younger patient tumors (2.2% at highest *vs <*0.1% in ≥65 group, adjusted *P* for trend = 0.02), followed by *IDH2* SNVs (2.2% at highest *vs* 0.1% in ≥65 group, adjusted *P* for trend = 0.15), *SMARCB1* SNVs (1.1% at highest *vs* 0.1% in ≥65 group, adjusted *P* for trend = 0.13), *IRF4* SNVs (1.1% at highest *vs* 0.1% in ≥65 group, adjusted *P* for trend = 0.23), and fusions with *FLI1* (1.3% at highest *vs* 0% in ≥65 group, *P* for trend=0.15). Decreases in the prevalence of *RBM10* and *DNMT3A* SNVs with age were dramatic, with the <65 age group having half the prevalence seen in the ≥65 group (OR = 0.43, adjusted *P* ≤ 1E-3), and no SNVs in either being detected in patients <50 years of age. These observations were expected for *DNMT3A* as alterations in this gene are commonly found in older individuals due to age-related clonal hematopoiesis (41).

#### 3.2.3 Gene-level genomic alterations and individual variants with differing prevalence between younger and older patient tumors when stratifying by sex

Of the 24 detected gene-level genomic alteration and individual variant associations, 14 (58%) were detected when analyzing female and/or male patient tumors separately (**Figures 1B, C**; **Table 2**). Genomic alteration associations detected in both female and male stratified analyses included associations with *ALK* and *ROS1* fusions, *RBM10* SNVs, and the specific *EML4-ALK* fusion. Associations with SNVs in *KRAS*, *RAD51B* (R47*), and *SMARCB1* were detected when analyzing males alone, while associations with SNVs in *MET*, *DNMT3A, LZTR1, IRF4, IDH2*, and *MET* exon 14 skipping mutations were detected when analyzing females alone. Associations with SNVs in three genes were detected when analyzing either male or female patient tumors, but not when analyzing all patient tumors. These included SNVs in *CDK12* (females; 0.3% in ≥65 group *vs* 2.4% at highest in younger groups, adjusted *P* for trend=0.16), *TET2* (females; 2.4% in ≥65 group *vs ≤*1% in younger groups, adjusted *P* for trend = 0.24), *RAD51* (males; 0.1% in ≥65 group *vs* 2.3% at highest in younger groups, adjusted *P* for trend = 0.24), and amplification of *FGF3* (males; 2.1% in ≥65 group vs 4.8% at highest in younger groups, adjusted P for trend = 0.25). Interestingly, as with *DNMT3A*, alterations in *TET2* are commonly found in older individuals due to age-related clonal hematopoiesis (41). Full results for stratified analysis can be found in **Supplementary Tables 2B, C**, **3B, C**.

Overall, these results indicate both younger and older patients are enriched for certain genomic alterations, with younger patient tumors being enriched for several genomic alterations in genes with known or potential clinical significance for NSCLC. Two of the genomic alterations found enriched in older patient tumors may be attributed to age-related clonal hematopoiesis (*DNMT3A, TET2*) (41), which may or may not be relevant to the actual tumor biology. Age associations with genomic alteration prevalence were detected at varying younger age group thresholds (**Table 2**, “Oldest age group first detected for”) and some were detected when analyzing female or male patient tumors only, showing heterogeneity in associations due to chosen age threshold and sex.

### 3.3 Distinct TMB and immune microenvironment profiles between tumors of younger and older patients

#### 3.3.1 TMB and immunogenic signature profiles of younger and older patient tumors

**Figure 2:**
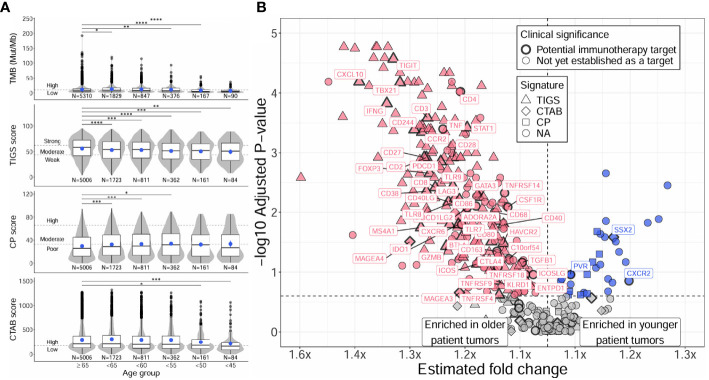
Distinct TMB, immunogenic signatures, and immune gene expression profiles between younger and older patients with NSCLC. Differences between younger and older patients with NSCLC were assessed for TMB and immunogenic signatures (TIGS, CP, CTAB) **(A)**, and individual immune gene expression **(B)**. Differences in younger age groups vs the ≥65 age group were assessed via Firth’s penalized logistic regression adjusted for tumor specimen site and histology. *P* for trends of decreasing measures with decreasing age were obtained from standard or penalized likelihood ratio tests as described in the Methods section. For plotting TMB, TIGS, CP, and CTAB data (left column), each individual tumor from a unique patient falling into a particular age group (x-axis) was plotted along the y-axis based on its TMB, TIGS, CP, or CTAB value. The bottom, middle, and top horizontal boundaries of each box in the box-violin plots represent the first, second (median), and third quartiles of the data for a particular age group. The lines extending from the two ends of each box represent 1.5x outside the interquartile range. Points beyond the lines are considered outliers. The width of the grey shaded regions around the boxes represents the density of the data points, where wider areas correspond to higher data point density. Blue points within each box-violin plot represent the mean of the data. Bars above the box-violin plots denote which age groups were detected as having significantly different distributions in TMB, TIGS, CP, or CTAB values, and the number of asterisks indicate the level of significance (* <0.05, ** <0.01, *** <0.001, **** <0.0001). For TMB, CP, and CTAB, although plotting was done using untransformed values, testing was performed on log transformed values. For plotting differential gene expression results (right column), 397 tested immune genes were plotted based on their estimated fold change (x-axis) and -log10 adjusted *P* for trend (y-axis). To calculate a single estimated fold change for plotting, coefficients of a gene from testing each age group against the ≥65 age group were averaged, then the exponent taken. Points were colored blue if gene expression was enriched in younger patient tumors, and dark pink if enriched in older patient tumors. Shapes of the points denote if a gene is used in calculating a particular immunogenic signature (triangle=TIGS, diamond=CTAB, square=CP, circle=NA, not assigned to an immunogenic signature). Points that correspond to significant immune genes with potential clinical significance are labeled with gene names and were given a bold border. TMB, tumor mutational burden; TIGS, tumor immunogenic signature; CP, cellular proliferation signature; CTAB, cancer testis antigen burden signature; P, *P* for trend from standard or penalized likelihood ratio tests.

Distinct TMB and immunogenic signature profiles were detected for younger and older patients, majorly characterized by a reduction of these measures with decreasing age (**Figure 2A**; **Supplementary Table 4A**). Both TMB and TIGS scores (signature for tumor inflammation) decreased significantly with decreasing age (*P* for trend < 2E-4) and were significantly lower in almost all age groups compared against the ≥65 group (**Figure 2A**, first and second rows, **Supplementary Table 4A**). Younger patient tumors also had reduced CTAB scores (signature for cancer testis antigen expression within a patient’s tumor) (*P* for trend = 0.03), although this was primarily driven by tumors from patients <50 years of age (**Figure 2A**, bottom row, **Supplementary Table 4A**). The only immunogenic signature to increase with decreasing age was CP score (signature for cellular proliferation), where CP scores increased slightly, but significantly, with decreasing age until 50 – 55 years, then leveled off (**Figure 2A**, third row, **Supplementary Table 4A**). Adjusting the analysis for presence or absence of genomic alterations in *ALK* or *EGFR* genes did not change overall results, only causing minor fluctuations in the significances of associations (**Supplementary Table 4A**).

#### 3.3.2 Differential immune gene expression between younger and older patient tumors

Differential gene expression analysis of individual immune gene transcripts reflected what was observed at the signature-level, with 61% (244/397) of tested immune-related genes having significantly lower expression in younger patient tumors compared to the ≥65 group (adjusted *P* for trend <0.25; **Figure 2B**; **Supplementary Table 5A**). Younger patient tumors were only enriched for 8% (33/397) of tested genes, the most significant of which were the genes *MYC*, *CXCL8* (*IL8*), *S100A8*, *HIF1A*, *S100A9, TOP2A, PYGL, BRCA2, SSX2, VEGFA*, and *LRG1* (adjusted *P* for trend <0.05). Similarly to the analyses of TMB and immunogenic signatures, adjusting for presence or absence of genomic alterations in *ALK* or *EGFR* genes did not change overall results; 7 genes lost significance, but 11 gained significance, which was caused by minor fluctuations in multiple testing corrected *P*-values (**Supplementary Table 5A**). Genes with higher expression in younger patient tumors were found to be enriched in GO biological processes concerning cellular proliferation, migration, and response to hypoxia (e.g., “positive regulation of epithelial cell proliferation”, fold enrichment >100, adjusted *P* = 1E-3; “positive regulation of chemotaxis”, fold enrichment = 88, adjusted *P* = 0.02; “response to hypoxia”, fold enrichment = 85.8, adjusted *P* = 4E-3), while genes with higher expression in older patient tumors were found to be enriched in biological processes concerning activation of both innate and adaptive immune responses (e.g., “natural killer cell activation”, fold enrichment = 83.7, adjusted *P* = 3E-5; “neutrophil activation”, fold enrichment = 55.8, adjusted *P* = 6E-3; “T cell activation involved in immune response”, fold enrichment = 55.8, adjusted *P* = 6E-3) (**Supplementary Table 6A**).

#### 3.3.3 Differences in high expression of immune markers with potential clinical significance as immunotherapy targets

Among the tested immune genes, expression of 64 (16%) have been previously validated for monitoring immune activity in solid tumors (30, 31) and could indicate potential immunotherapy targets involved in clinical trial inclusion criteria. Of these genes, expression of 48 (75%) were detected as having significantly lower expression in younger patient tumors compared to the ≥65 group (adjusted *P* for trend <0.25; **Figure 2B**; **Supplementary Table 5A**). Younger patient tumors were only enriched for 3 (5%) of these genes including one cancer testis antigen gene (*SSX2*) and two genes involved in T-cell recognition and killing of cancer cells (*PVR*, *CXCR2*). Similarly, when testing for differences between younger and older patient tumors in the prevalence of “high expressing” tumors (normalized gene expression rank ≥75% when compared to pan-tumor control samples) for each of the 64 immune genes, younger patient tumors were less likely to have high expression for 44 genes (69%) and more likely to have high expression for only 2 genes (3%; *CXCR2*, *SSX2*) (**Supplementary Table 7A**).

#### 3.3.4 TMB, immunogenic signatures, and immune gene expression profiles of younger and older patient tumors when stratifying by sex

**Figure 3:**
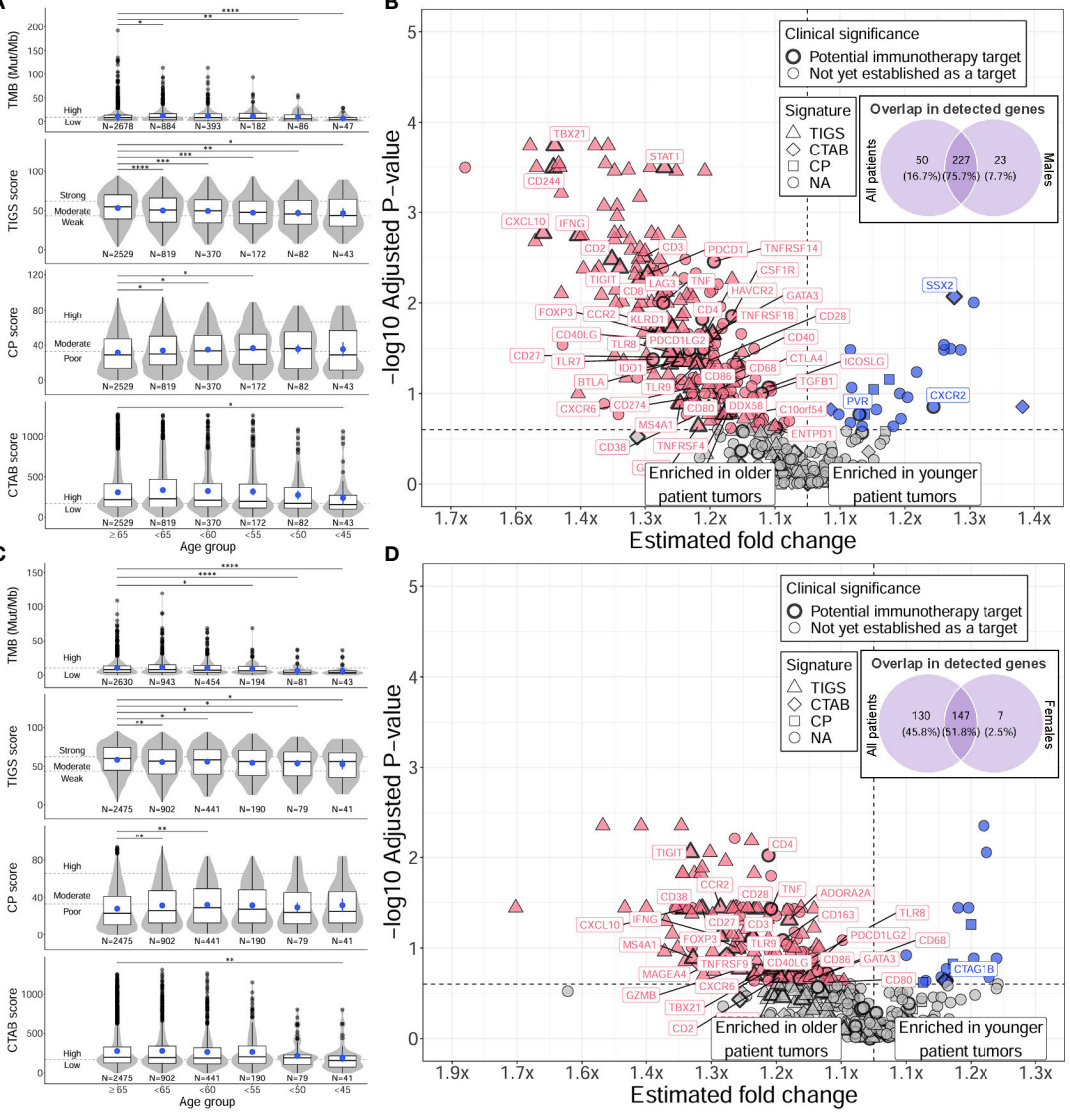
Distinct TMB, immunogenic signatures, and immune gene expression profiles between younger and older male and female patients with NSCLC. For males **(A, B)**, and females **(C, D)**, differences between younger and older patients with NSCLC were assessed for TMB and immunogenic signatures (TIGS, CP, CTAB) **(A, C)**, and individual immune gene expression **(B, D)**. Differences in younger age groups vs the ≥65 age group were assessed via Firth’s penalized logistic regression adjusted for tumor specimen site and histology. *P* for trends of decreasing measures with decreasing age were obtained from standard or penalized likelihood ratio tests as described in the Methods section. For plotting TMB, TIGS, CP, and CTAB data (left column), each individual tumor from a unique patient falling into a particular age group (x-axis) was plotted along the y-axis based on its TMB, TIGS, CP, or CTAB value. The bottom, middle, and top horizontal boundaries of each box in the box-violin plots represent the first, second (median), and third quartiles of the data for a particular age group. The lines extending from the two ends of each box represent 1.5x outside the interquartile range. Points beyond the lines are considered outliers. The width of the grey shaded regions around the boxes represents the density of the data points, where wider areas correspond to higher data point density. Blue points within each box-violin plot represent the mean of the data. Bars above the box-violin plots denote which age groups were detected as having significantly different distributions in TMB, TIGS, CP, or CTAB values, and the number of asterisks indicate the level of significance (* <0.05, ** <0.01, *** <0.001, **** <0.0001). For TMB, CP, and CTAB, although plotting was done using untransformed values, testing was performed on log transformed values. For plotting differential gene expression results (right column), 397 tested immune genes were plotted based on their estimated fold change (x-axis) and -log10 adjusted *P* for trend (y-axis). To calculate a single estimated fold change for plotting, coefficients of a gene from testing each age group against the ≥65 age group were averaged, then the exponent taken. Points were colored blue if gene expression was enriched in younger patient tumors, and dark pink if enriched in older patient tumors. Shapes of the points denote if a gene is used in calculating a particular immunogenic signature (triangle=TIGS, diamond=CTAB, square=CP, circle=NA, not assigned to an immunogenic signature). Points that correspond to significant immune genes with potential clinical significance are labeled with gene names and were given a bold border. Venn diagrams within each plot represent the overlap of differentially expressed genes between male or female only analyses and analysis of all patients. TMB, tumor mutational burden; TIGS, tumor immunogenic signature; CP, cellular proliferation signature; CTAB, cancer testis antigen burden signature; P, *P* for trend from standard or penalized likelihood ratio tests.

When analyzing tumor characteristics separately for male and female patients, distinct patterns emerged in the associations of age with TMB, immunogenic signatures, and immune gene expression (**Figure 3**). In males, results for TMB, TIGS, and CP closely resembled those of all patients, with decreasing TMB, TIGS, and CP as age decreased (*P* for trend = 2E-5 to 0.05) and there was a large overlap in differentially expressed genes (76%) and immune markers with differential prevalence of high expression (78%) between younger and older male patient tumors (**Figures 3A, B**; **Supplementary Tables 4C**, **5C**, **7C**). However, the association between decreasing age and decreasing CTAB score was not significant (*P* for trend = 0.19) (**Figure 3A**, bottom row). In females, results for TMB and all immunogenic signatures (including CTAB score) mirrored those from analyzing all patients (*P* for trend = 2E-3 to 0.08) (**Figure 3C**; **Supplementary Table 4B**), however, the association between decreasing age and decreasing TIGS score was two orders of magnitude less significant than that observed for males (*P* for trend = 2E-3 *vs* 2E-5). Additionally, there was a substantial reduction in differentially expressed immune genes between younger and older patient tumors (39% of tested genes) compared to males (63% of tested genes) and all patients (70% of tested genes). The number of immune markers with differential prevalence of high expression was also substantially reduced in female patient tumors (33% of tested genes) compared to males (70% of tested genes) and all patients (72% of tested genes). However, higher prevalence of *CXCR2* high expressing tumors in younger patient tumors was robust across male and female tumors (37% at highest *vs* ~22% in ≥65 group, adjusted *P* for trend ~ 0.1; **Supplementary Tables 7B, C**).

#### 3.3.5 Differences in TMB, immunogenic signatures, and immune gene expression between males and females

**Figure 4:**
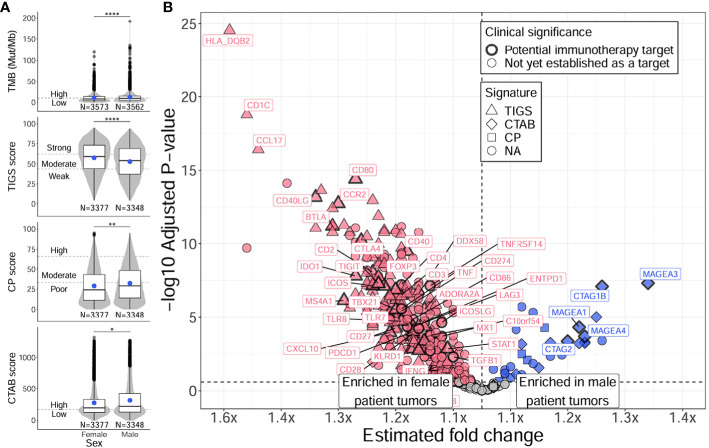
Distinct TMB, immunogenic signatures, and immune gene expression profiles between female and male patients with NSCLC. Differences between female and male patients with NSCLC were assessed for TMB and immunogenic signatures (TIGS, CP, CTAB) **(A)**, and individual immune gene expression **(B)**. Differences between female and male patient tumors were assessed via Firth’s penalized logistic regression adjusted for tumor specimen site and histology. For plotting TMB, TIGS, CP, and CTAB data (right column), each individual tumor from a unique male or female patient (x-axis) was plotted along the y-axis based on its TMB, TIGS, CP, or CTAB value. The bottom, middle, and top horizontal boundaries of each box in the box-violin plots represent the first, second (median), and third quartiles of the data for females or males. The lines extending from the two ends of each box represent 1.5x outside the interquartile range. Points beyond the lines are considered outliers. The width of the grey shaded regions around the boxes represents the density of the data points, where wider areas correspond to higher data point density. Blue points within each box-violin plot represent the mean of the data. Bars above the box-violin plots denote significantly different distributions in TMB, TIGS, CP, or CTAB values, and the number of asterisks indicate the level of significance (* <0.05, ** <0.01, **** <0.0001). For TMB, CP, and CTAB, although plotting was done using untransformed values, testing was performed on log transformed values. For plotting differential gene expression results (right column), 397 tested immune genes were plotted based on their estimated fold change (x-axis) and -log10 adjusted *P*-value (y-axis). Points were colored blue if gene expression was enriched in male patient tumors, and dark pink if enriched in female patient tumors. Shapes of the points denote if a gene is used in calculating a particular immunogenic signature (triangle=TIGS, diamond=CTAB, square=CP, circle=NA, not assigned to an immunogenic signature). Points that correspond to significant immune genes with potential clinical significance are labeled with gene names (as space allowed) and were given a bold border. Additionally, genes that reached adjusted *P* < 1E-15 are also labeled. TMB, tumor mutational burden; TIGS, tumor immunogenic signature; CP, cellular proliferation signature; CTAB, cancer testis antigen burden signature.

To discern if the variability in age associations observed in male and female only analyses might be due to differences in TMB and immune-related measurements between males and females, we tested for differences in these metrics between males and females regardless of age (**Figure 4**; **Supplementary Tables 4D**, **5D**). When comparing female and male patient tumors, females displayed significantly higher TIGS overall (*P* = 1E-12), indicating the presence of increased inflammation compared to male patient tumors (**Figure 4A**, second row, **Supplementary Table 4D**). Male patient tumors had significantly higher scores in all other metrics (TMB, CP, CTAB; *P* = 4E-5 – 0.04) (**Figure 4A**; **Supplementary Table 4D**). A substantial majority of tested immune genes (79%), including almost all of the 64 immune markers with potential clinical significance (94%), showed significantly higher expression in female tumors compared to males, while only a small proportion of tested genes (8%) exhibited higher expression in male tumors, particularly genes that are included in CTAB score calculation (*MAGEA* genes, *MAGEC2*, *CTAG1B*, *CTAG2*, *GAGE* genes, and *BAGE*) (**Figure 4B**; **Supplementary Table 5D**), which aligns with significantly increased levels of CTAB scores observed in male tumors (**Figure 4A**, bottom row, **Supplementary Table 4D**). Like older patients, genes with higher expression in female patient tumors were found to be enriched in GO biological processes concerning activation of both innate and adaptive immune responses (e.g., “natural killer cell activation”, fold enrichment = 65.2, adjusted *P* = 6E-5; “positive regulation of T cell proliferation”, fold enrichment = 45.1, adjusted *P* = 1E-12; “neutrophil activation”, fold enrichment = 43.4, adjusted *P* = 8E-3) (**Supplementary Table 6B**). Genes with higher expression in male patient tumors were enriched in biological processes concerning metabolic processes and cell cycle control (e.g., “tRNA threonylcarbamoyladenosine metabolic process”, fold enrichment > 100, adjusted *P* = 0.02; “mitotic cell cycle phase transition”, fold enrichment = 32.9, adjusted *P* = 0.02; “cell cycle phase transition”, fold enrichment = 32.9, adjusted *P* = 0.02) (**Supplementary Table 6B**).

Together, these results indicate younger patient tumors have distinct TMB and immune microenvironment profiles from older patient tumors characterized by lower TMB and immune system activation and higher cellular proliferation and migration with a more hypoxic tumor microenvironment. These observations are recapitulated in male patient tumors; however, female patient tumors show less of an association between age and immune activation-based gene expression, which may be due to overall higher levels of immune activation in female patient tumors, regardless of age (**Figure 4**).

### 3.4 Effect of age in combination with sex on immunotherapy outcomes

**Figure 5:**
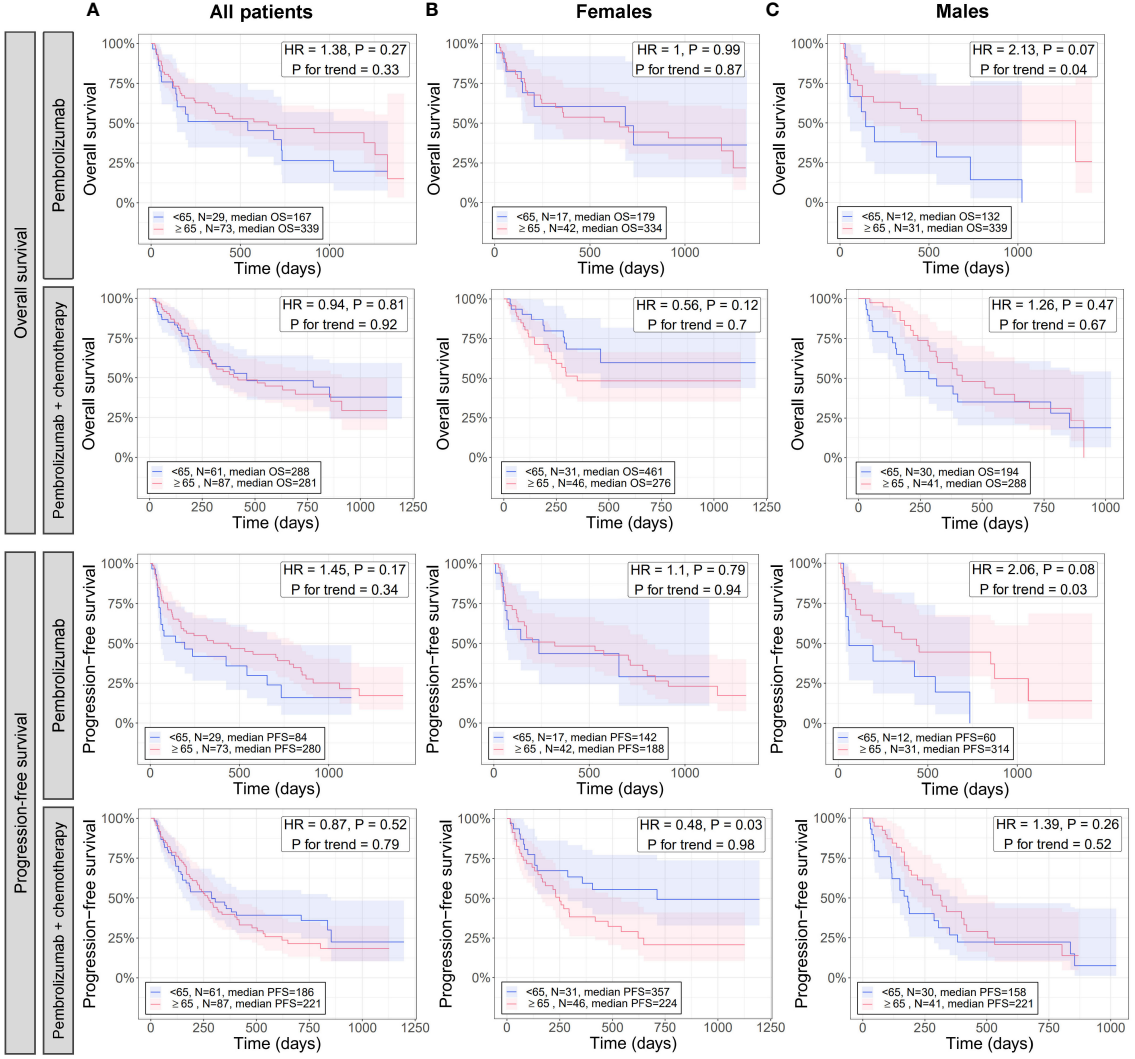
Effect of age in combination with sex on the overall and progression free survival of patients with NSCLC treated with pembrolizumab immunotherapy and pembrolizumab immunotherapy with chemotherapy. For all patients **(A)**, females **(B)**, and males **(C)**, differences between younger and older patients with NSCLC in overall (rows 1 and 2) and progression free (rows 3 and 4) survival when treated with pembrolizumab (rows 1 and 3) or pembrolizumab with chemotherapy (rows 2 and 4) were assessed via Cox proportional hazards regression (adjusted for histology) and Kaplan-Meier curves. Cox regression was performed once for testing survival differences between NSCLC patients <65 or ≥65 years and again testing association of decreasing age with survival (*P* for trend). Kaplan-Meier curves were created only for the <65 and ≥65 age groups as these had the largest N and statistical power. HR, hazard ratio from Cox regression testing differences in survival between <65 and ≥65 age groups; *P*, the *P*-value of the HR.

Results from analyzing immunogenic signatures and individual immune gene expression suggests younger patients (especially males) have less immunogenic tumors compared to older patients (**Figures 2**, **3**). To evaluate whether differences in tumor immune microenvironment translate to differential responses of patients to immunotherapy, we evaluated the association of age with OS and PFS in an independent cohort of patients with NSCLC who were treated with either pembrolizumab or pembrolizumab with chemotherapy (**Figure 5**).

When analyzing all patients, there were no significant differences in OS and PFS between patients <65 and ≥65 years for either therapy (HR = 0.9 to 1.4, *P* ≥ 0.17), and no trends for association of survival with decreasing age were detected (*P* for trend ≥ 0.3) (**Figure 5A**). Similarly, when analyzing only female patients, no significant differences in OS between patients <65 and ≥65 years were observed for either therapy (HR = 0.6 to 1, *P* ≥ 0.1) (**Figure 5B**, first and second rows), or for PFS of patients treated with pembrolizumab (HR = 1.1, *P* = 0.79) (**Figure 5B**, third row). However, a significant difference in PFS was detected between patients <65 and ≥65 years for those on pembrolizumab with chemotherapy (HR = 0.5, *P* = 0.03) where female patients <65 years had significantly longer PFS than those ≥65 years (**Figure 5B**, fourth row). No trends for association of survival with decreasing age were detected when analyzing only female patients (*P* for trend ≥ 0.7) (**Figure 5B**). When analyzing male patients given pembrolizumab, no significant differences in OS and PFS between patients <65 and ≥65 years (HR = 1.3 to 1.4, *P* ≥ 0.3), and no trends for association of survival with decreasing age (*P* for trend ≥ 0.5) were detected (**Figure 5C**, second and fourth rows). However, borderline-significant differences in OS and PFS between patients <65 and ≥65 years (HR = 2.1, *P* ≤ 0.08), and significant associations of decreased survival with decreasing age (*P* for trend ≤ 0.04) were detected when analyzing male patients on pembrolizumab alone (**Figure 5C**, first and third rows). No significant differences in OS or PFS were found between female and male patients overall, regardless of age (*P* > 0.05; **Supplementary Figure 1**), which has also been noted in previous studies (42).

Overall, these results indicate younger male patients (shown in our previous analysis to have less tumor immune activation) have significantly worse outcomes than older male patients when given immunotherapy alone, but have similar outcomes when chemotherapy is added. Contrarily, younger and older female patients (shown in our previous analysis to have lesser differences in tumor immune activation than males) have similar outcomes on immunotherapy alone, but younger female patients (<65 years) had significantly better outcomes than older female patients when chemotherapy is added. Altogether, these results, in conjunction with results from our previous analysis, suggest underlying age- and sex-associated differences in the tumor immune microenvironment may play a role in differential response of patients to immunotherapy.

## 4 Discussion

In summary, we performed a retrospective analysis of 8,230 patients with NSCLC tested by CGIP during routine clinical care. We compared the landscape of genomic alterations and the tumor immune microenvironment of younger and older patients with NSCLC, taking differences between male and female patients into account. We defined an older patient group using a real-world relevant age threshold and using a 5-year sliding threshold from <45 to <65 years to define various thresholds of younger patients. We used an independent cohort of patients with immunotherapy outcomes data to test the combinatorial effect of age and sex on OS and PFS of patients who received immunotherapy or immunotherapy with chemotherapy. We observed distinct genomic and immune microenvironment profiles for tumors of younger patients compared to tumors of older patients. Younger patient tumors were enriched in clinically relevant genomic alterations and had gene expression patterns indicative of reduced immune system activation, which was most evident when analyzing male patients. Further, we found younger male patients treated with immunotherapy alone had significantly worse survival compared to male patients ≥65 years, while the addition of chemotherapy reduced this disparity. Contrarily, we found younger female patients had significantly better survival compared to female patients ≥65 years when treated with immunotherapy plus chemotherapy, while treatment with immunotherapy alone resulted in similar outcomes. To our knowledge, this is the first study to comprehensively investigate differences in genomic alteration prevalence, tumor immune microenvironment, and responses to immunotherapy in parallel within the same study. This helps to create a more complete view of the genomic and immune landscape of younger patient tumors and how it influences response to immunotherapy.

One strength of our study is the novel way in which we defined the younger patients with NSCLC. Most age-related NSCLC studies to date use an analysis approach where a single age cutoff is selected (typically 40 to 50 years) to classify younger patients, and then compared that singularly defined group against older patients (3, 6, 7, 9–13). This analytical approach poses inherent biases based on the age threshold chosen to define younger and older patients as shown by the various age thresholds at which an association was detected in the current study (**Table 2**, column “Oldest age group first detected for”). In contrast, our study adopted a more comprehensive approach by using a sliding window of 5 years to create multiple groups of younger patients from <45 to <65, testing each group against the older patient reference group, and testing for trends across all younger patient ages to detect features that associate with decreasing age. This analytical approach enabled us to conduct comparisons across a spectrum of younger ages, therefore, not committing to a single threshold to define young patients. The size of the study population (>8,000 patients) allowed us to implement this analytical approach, giving us sufficient statistical power to detect statistically significant differences in all age groups, even when stratifying by males and females. In the results, we highlighted features that reached significance when testing how it trended with decreasing age, however, all test results for each age group are presented in the **Supplementary Tables**. Bias in the analyses is still introduced by a single threshold chosen for the older patient group (≥65 years). Multiple thresholds for both younger and older patients would vastly reduce the power of the analyses, requiring much larger samples sizes, and increase the difficulty of interpreting results, therefore, we opted for one threshold for older patients that reflects a real-world threshold with consequences for clinical testing in the US.

Using our analytical approach, we showed that tumors of younger patients with NSCLC were enriched for genomic alterations in key NSCLC driver-genes including *EGFR*, *ALK*, *ROS1*, and *NTRK1*. Specific, clinically significant alterations with an associated FDA-approved targeted therapy were detected as enriched in younger patient tumors including two *EGFR* deletions, E746-A750 and L747-E749, and an *ALK* fusion, *EML4-ALK*. These observations align with a growing body of research conducted across various geographical regions worldwide (3, 6, 7, 9–13). We showed that younger patient tumors had significantly lower prevalence of certain genomic alterations including *KRAS* SNVs and *MET* exon 14 skipping mutations, which have previously been reported to be associated with older age (3, 13, 43).

Younger patient tumors displayed enrichment of several other potentially clinically significant genomic alterations that were classified as having FDA-approved therapies in other tumor types or that are part of a clinical trial inclusion for NSCLC. These included SNVs in genes *RAD51B*, *SMARCB1*, *IDH2*, *CDK12* (females only), *RAD51* (males only), amplification of *FGF3* (males only), and specific substitutions in *RAD51B* (R47*) and *EGFR* (A750P). Patients with prostate cancer with certain *RAD51B* or *CDK12* SNVs, including *RAD51B* R47*, are FDA-approved for treatment with olaparib (a PARP inhibitor). Certain SNVs in *SMARCB1* and *IDH2* and amplification of *FGF3* were included in inclusion criteria for several clinical trials at the time of testing including trials of olaparib (*IDH2*) (44), atezolizumab + tiragolumab (*SMARCB1*) (45), and other therapies. Patients with the specific *EGFR* A750P variant, in combination with the E746-A750 or L747-E749 deletion, were candidates for a clinical trial of patritumab deruxtecan (HER3-Dxd; a novel, investigational, HER3-directed antibody-drug conjugate) in combination with osimertinib (a tyrosine kinase inhibitor) (46). Of these alterations, enrichment of genomic alterations in *EGFR* (3, 21) have been noted previously in tumors of younger patients. Alterations in the genes *ERBB2* and *RET* have been previously reported as being enriched in younger patient tumors (5, 18, 47), but were not detected as such in the current study. This may be due to differences between the current study and previous studies including differences in analytical approach, underlying populations (majority of *ERBB2* and *RET* associations detected in East Asian populations), and others.

We detected multiple genomic alterations that were enriched in younger patient tumors and were not classified as currently having an associated FDA-approved therapy for other tumor types, clinical trial inclusion criteria for NSCLC, or other evidence for plausible therapeutic significance for NSCLC. These alterations included SNVs in the genes *CREBBP, LZTR1*, *IRF4*, and fusions with *FLI1*. *CREBBP* encodes an acetyltransferase and is one of the most mutated genes in small cell lung cancer (48), however, co-mutations in this gene and *NOTCH1* might negatively affect the benefit of adjuvant therapy in *EGFR-*mutated NSCLC tumors (49). *LZTR1* encodes a protein that regulates polyubiquitination and degradation of RAS proteins and has been suggested to regulate the growth and invasion of lung adenocarcinoma cells through RAS/MAPK signaling (50). *IRF4* encodes a transcription factor important in regulating immune responses and has been shown to be overexpressed in NSCLC tumor tissue and may play a role in increasing cell proliferation rate and colony formation of NSCLC tumor cells through activation of the Notch-Akt signaling pathway (51). *FLI1* also encodes a transcription factor whose high expression was found to be a poor prognostic factor in NSCLC (52). Although not yet annotated as clinically relevant to NSCLC or other cancer types, evidence exists for their emerging importance in the progression of NSCLC.

Immunotherapy has become a mainstay of treatment in NSCLC; however, it is crucial to verify the presence of oncogenic driver alterations and biomarkers for response to immunotherapy prior to initiating treatment (53). Patients with oncogenic driver alterations and less immunogenic tumors derive minimal therapeutic benefit from immunotherapy, both of which we observed here for younger patients with NSCLC, especially younger male patients. Consistent with previous reports, we showed younger patients with NSCLC present with lower TMB and differentially expressed immune-related genes compared to older patients (7, 14), and that these observations were not driven by the presence or absence of underlying genomic alterations in key NSCLC driver genes *ALK* or *EGFR*. Further, we revealed younger patient tumors had markedly reduced gene expression related to activation of the innate and adaptive immune system, and that this reduction was most evident in male patients. This trend was also detected when looking at high expression of a subset of immune markers with clinical potential to be immunotherapy targets. When analyzing tumors from male and female patients separately, immunogenic signatures within female tumors had much less of an association with age due to their tumors having higher immune system activation overall. These findings suggested that younger patients with NSCLC, especially males, would see less benefit from treatment with immunotherapy. We tested this in an independent cohort of NSCLC patients who previously underwent treatment with pembrolizumab or pembrolizumab with chemotherapy. We observed younger male patients with NSCLC had shorter OS and PFS when treated with pembrolizumab alone, yet survival between younger and older patients were similar when adding chemotherapy. Younger female patients had similar outcomes to older female patients without the addition of chemotherapy but had longer PFS when chemotherapy was added. These observations resonate with what was observed in the main discovery cohort and suggests overall that younger male patients with NSCLC have less response to immunotherapy alone due to their “colder” tumor immune microenvironment unlike female patients with NSCLC who respond equally to immunotherapy across all ages due to having “hot” tumor immune microenvironments regardless of age. This combinatorial effect of age and sex on immunotherapy response may be one factor that has led to inconsistent findings between previous studies investigating the effect of age on immunotherapy response in NSCLC (22–26), and proves to be an important factor when considering treatment options for younger patients with NSCLC.

Although the current study was designed to be as comprehensive and statistically rigorous as possible, notable limitations were still present including lack of smoking history information and the small sample size of patients <50 years. As stated previously, this study is a retrospective analysis of real-world clinical testing data obtained from CGIP performed during a patient’s standard care, therefore, only data pertinent to completing testing was obtained when testing was ordered. While key variables such as age, sex, diagnosis, histology, tumor specimen location, and others are collected, environmental and lifestyle exposures, such as smoking history, are not. Still, steps were taken to reduce the effect of smoking history on results by ensuring no significant differences existed between younger and older patient groups for smoking-associated variables (i.e., sex), and if differences were detected (i.e., tumor histology), adjusting for them during analyses. In terms of sample size, while the overall number of cases included in the present study was high (>8,000), the number of patients <50 years were less than 200, hence this study may not have been powered to detect certain genomic and immune-related associations that require higher sample size for this group of patients. The low sample size of this group also did not allow for stratification by genomic alteration presence when analyzing immune-related data or outcomes data, which would allow further characterization based on underlying genomic characteristics. Real-world CGIP testing data of NSCLC is biased towards older patients with advanced disease as this patient group is typically covered by insurance to receive CGIP testing. Finally, while the same assay was used to assess immune gene expression of patient tumors for both the main discovery cohort and the independent outcomes cohort (Oncomine™ Immune Response Research Assay), different assays were used for genomic profiling, hence, genomic results could not be compared across cohorts as the assay used for profiling the independent outcomes cohort targeted less genes than that used for the main discovery cohort (144 *vs* 523).

Comprehensive genomic and immune profiling serves as a cornerstone for the emerging field of personalized medicine, allowing healthcare providers to customize treatment plans based on the unique makeup of each patient’s tumor genomics and immune microenvironment (54). We show here that tumors of younger patients with NSCLC are enriched for oncogenic driver alterations, are less immunogenic overall, and are less likely to be high expressors of immunotherapy targets, although this was not the case for all tested markers (e.g., *CXCR2*). These observations suggest younger patients should be tested via CGIP to aid therapy decision making in finding more precise targeted therapies or immunotherapies. Medicare, a US-based government program that provides health insurance for people aged 65 and older, typically covers CGIP for older patients. Younger patients with NSCLC often face challenges with commercial insurance coverage for tissue-based genomic and immune profiling even though we (in the current study) and others have shown evidence that younger patients may have increased success of matching to a targeted therapy or immunotherapy based on their enrichment of targetable genomic alterations or specific immune markers. Our study reiterates the importance of CGIP in younger patients with NSCLC by showing the important implications it has for the successful treatment of NSCLC in younger patients. Expanding coverage of CGIP for younger NSCLC patients would support greater access to genomic and immune profiling, potentially increasing the chance of matching younger NSCLC patients to effective therapies.

## Data availability statement

De-identified, individual-level patient data, genomic variants, and individual immune gene expression data used in the article can be found on Zenodo (https://zenodo.org/record/11396552). An R markdown file with R code used to perform the analyses and generate figures is also provided in the repository along with the data. All versions of software used are provided in the Methods section of the article. Raw sequencing data were derived from routine clinical testing of real-world patients and cannot be shared publicly. Data for immune gene expression signatures are not publicly available due to a non−provisional patent filing covering the methods used to generate and analyze these data but are available from the corresponding author on reasonable request.

## Ethics statement

The studies involving humans were approved by Western Institutional Review Board Copernicus Group (WCG protocol # 1340120). The studies were conducted in accordance with the local legislation and institutional requirements. The human samples used in this study were acquired from a by- product of routine care or industry. Written informed consent for participation was not required from the participants or the participants’ legal guardians/next of kin in accordance with the national legislation and institutional requirements.

## Author contributions

ZW: Conceptualization, Data curation, Formal analysis, Investigation, Methodology, Visualization, Writing – original draft, Writing – review & editing. HK: Conceptualization, Writing – original draft, Writing – review & editing. MN: Conceptualization, Resources, Supervision, Writing – original draft, Writing – review & editing. SH: Project administration, Writing – review & editing. KS: Writing – review & editing. RP: Writing – original draft, Writing – review & editing. SZ: Data curation, Methodology, Resources, Writing – review & editing. SP: Data curation, Methodology, Resources, Writing – review & editing. JC: Data curation, Methodology, Resources, Writing – review & editing. JJ: Writing – review & editing. KS: Writing – review & editing. TJ: Writing – review & editing. ME: Resources, Supervision, Writing – review & editing. BC: Resources, Supervision, Writing – review & editing. PS: Writing – review & editing. ES: Resources, Supervision, Writing – original draft, Writing – review & editing. SR: Resources, Supervision, Writing – review & editing.
