# Supplementary material for: Real-world comprehensive genomic and immune profiling reveals distinct age- and sex-based genomic and immune landscapes in tumors of patients with non-small cell lung cancer
Source: Front Immunol. 2024 Jun 21;15:1413956. doi: 10.3389/fimmu.2024.1413956 (PMC11224431; doi:10.3389/fimmu.2024.1413956)
Supplement: Supplementary file 8 [file Image_1.pdf]

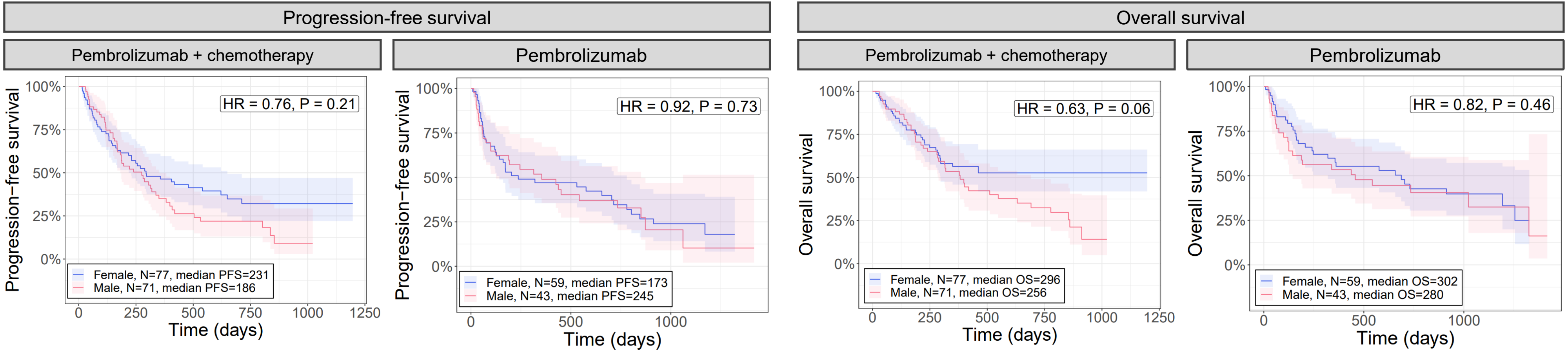

Supplementary Figure 1. Differences between sex in the overall and progression-free survival of patients with NSCLC treated with pembrolizumab immunotherapy and pembrolizumab immunotherapy with chemotherapy. For all patients, differences between female and male patients with NSCLC in overall and progression-free survival when treated with pembrolizumab or pembrolizumab with chemotherapy were assessed via Cox proportional hazards regression (adjusted for histology) and Kaplan-Meier curves. HR, hazard ratio from Cox regression testing differences in survival between female and male groups; *P*, the *P*-value of the HR.
